# Chromatographic Separation of Phenolic Compounds from Extra Virgin Olive Oil: Development and Validation of a New Method Based on a Biphenyl HPLC Column

**DOI:** 10.3390/ijms20010201

**Published:** 2019-01-08

**Authors:** Miguel D. Ferro, Sónia A. O. Santos, Armando J. D. Silvestre, Maria F. Duarte

**Affiliations:** 1 Centro de Biotecnologia Agrícola e Agro-Alimentar do Alentejo (CEBAL)/Instituto Politécnico de Beja (IPBeja), 7801-908 Beja, Portugal; miguel.ferro@cebal.pt; 2 CICECO—Aveiro Institute of Materials, Department of Chemistry, University of Aveiro, 3810-193 Aveiro, Portugal; santos.sonia@ua.pt (S.A.O.S.); armsil@ua.pt (A.J.D.S.); 3 Instituto de Ciências Agrárias e Ambientais Mediterrânicas (ICAAM), Universidade de Évora, Pólo da Mitra, 7002-554 Évora, Portugal

**Keywords:** extra virgin olive oil, hydrophilic phenolic compounds, chromatographic resolution, Kinetex biphenyl core-shell column, method validation

## Abstract

Three different high performance liquid chromatography columns were accessed for phenolic compounds (PC) separation in the hydrophilic fraction of extra virgin olive oil (EVOO). Two fully porous C_18_ bonded silica phases and one partially porous biphenyl column were used. Biphenyl column allowed for an increase of more than 30% in peak capacity (n_c_), higher selectivity (α) (1.045), and improved retention (k), with a reduction of 22.1% in the retention time. The higher resolution (R_s_) was obtained by using the biphenyl column, with a fair separation of oleuropein aglycone isomers (OAI) and a good identification of caffeic acid (CA). Tyrosol (T), hydroxytyrosol (HT), and dihydroxyphenyl glycol (DHPG) were also well separated and identified. Moreover, the method using a biphenyl column was fully validated according to the requirements for new methods. For all parameters, the method applying the biphenyl column proved to be a reliable, accurate, and robust tool for separation, identification, and quantification of the main PCs in EVOOs.

## 1. Introduction

Extra virgin olive oil (EVOO), which represents the primary source of fat intake in the Mediterranean diet, is well known for its health benefits, such as the protection of low density lipoprotein particles from oxidative damage, the maintenance of normal blood high density lipoprotein cholesterol concentrations, the maintenance of normal blood pressure, and its anti-inflammatory properties, among many others [1]. Phenolic compounds (PC) are among these health-promoters that are present in EVOO [2,3,4,5,6], which were highlighted by the European Food Safety Authority in 2012 with a health claim for virgin olive oil PCs, which contribute to the protection of blood lipids from oxidative stress [7]. Olive oil (OO) PCs are essential quality biomarkers; in particular, hydroxytyrosol (HT), the target of a health claim, which to be valid, requires the presence of a minimum of 5 mg of HT and its derivatives, per 20 g of OO. The health claim specifications prompted the development of new methods for the better identification and quantification of hydrophilic PCs present in EVOO [8,9].

Some of the PCs present in EVOO, particularly hydrophilic phenols, are specific from this source [10]. Despite being minor compounds, representing about 2wt % of EVOOs, hydrophilic phenols, such as phenolic acids, phenolic alcohols, hydroxyl-isochromans, flavonoids, secoiridoids, and lignans, play a key role on the EVOO quality and shelf-life, due to their antioxidant activity [10], with a large impact on the final organoleptic characteristics [11,12,13].

**Figure 1:**
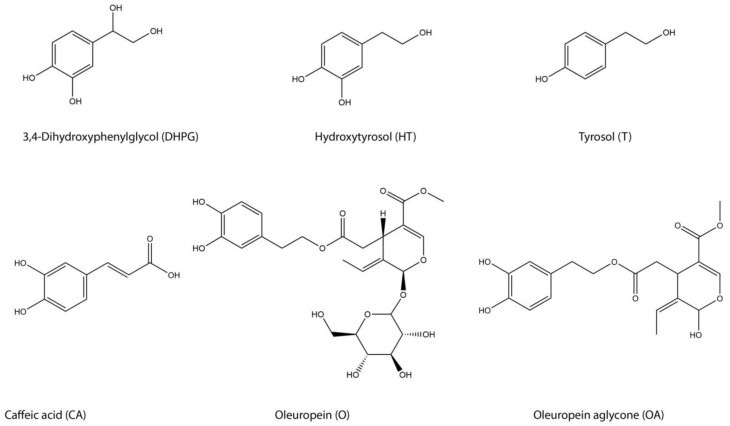
Identified phenolic compounds (PCs) in the hydrophilic fraction of extra virgin olive oil (EVOO).

The main phenolic alcohols that are present in EVOOs are 3,4-dihydroxyphenylethanol, and p-hydroxyphenyl ethanol, also known as HT, and tyrosol (T), respectively (Figure 1) [14]. These compounds result from secoiridoid aglycones hydrolysis, which are derived mainly from oleuropein degradation during EVOO storage [15]. Dihydroxyphenyl glycol (DHPG) and caffeic acid (CA) are present in lower amounts in EVOO, but they are also of particular interest, due to their high antioxidant activities [16,17,18]. The identification and quantification of these individual PCs in EVOO has been performed with a wide variety of analytical techniques [19]. High performance liquid chromatography (HPLC) currently represents the most popular and reliable technique for the analysis of these compounds, with a wide variety of detectors that are coupled with this system, with ultraviolet (UV) being the most common one [19,20], applying reverse-phase (RP) chromatography.

In RP chromatography, the most popular stationary phases consist of a fully porous non-polar silica-based octadecyl (C_18_)-bonded phase [19]. However, the need for improved selectivity and a wider working range of conditions (such as pH and aqueous mobile phase percentage) potentiated the emergence of new silica-based stationary phases, such as polar-embedded and polar-endcapped stationary phases [21]. These new silica-based stationary phases present modifications on the classical C_18_ phase, with the addition of a polar functional group in the alkyl chain, or an endcapping agent, respectively. The advantages of the stationary phase modifications described, such as working under a higher water content in the mobile phase, improved peak shape, and increased selectivity, triggered the attention of the researchers [22]. Furthermore, the successful performance of is attributed to lower tailing and retention for basic compounds at a neutral pH and at different selectivities, due to the presence of polar groups, which minimize undesirable interactions with residual silanols [21]. With improved efficiencies, the importance of faster separations had also been taking precedence in recent years. Higher flow rates reduce the run time, but also the efficiencies, due to resistance of mass transfer. One way to overcome this problem is through the incorporation of nonporous particles surrounded by a thin porous shell (core-shell particles), providing a reduced path for analytes to travel into the stationary phase, when compared to fully porous particles, within an acceptable backpressure [23].

The increasing demand for chromatographic resolution improvements has potentiated the appearance of new RP column chemistry, which are able to better resolve, and therefore identify/quantify, minor compounds from EVOOs, such as phenolic alcohols like HT and its derivatives.

According to our knowledge, no previous studies have been reported where a biphenyl core-shell column is applied for the separation and identification of hydrophilic PCs. Previous reports comparing HPLC columns for EVOO PCs separation have been published [24], but only considering conventional C_18_ columns. In this work, a biphenyl core-shell (Kinetex^®^) column was compared with two conventional C_18_-packed columns (LiChrospher and Spherisorb) for the separation and identification of EVOO hydrophilic PCs, with applications on a different set of samples. The results revealed an improved peak capacity, retention factor, selectivity, peak asymmetry and overall resolution when using the biphenyl core-shell column, and this was the method that was fully validated according to the requirements for the new methods.

## 2. Results and Discussion

### 2.1. Column Comparison for Peak Identification

The use of C_18_ columns for olive oil PCs separation, identification, and quantification has been extensively reported in the literature [19,20], but little is known about the use of the biphenyl phases on the analyses of these specific EVOO compounds.

Three columns were tested and compared for the separation and quantification of EVOO PCs, a Kinetex core-shell biphenyl stationary phase column, and two columns packed with conventional fully porous C_18_ stationary phase (LiChrospher RP18 and Spherisorb ODS2). In total, six EVOO hydrophilic phenolic compounds (Figure 1) were separated and identified, applying liquid–liquid extraction (LLE) of EVOO, as it is reported to be the simplest andmost rapid, efficient, and cost-effective technique, when compared to solid phase extraction (SPE) [25]. Among the identified compounds were some common PCs of olive oil, namely oleuropein (O), oleuropein aglycone (OA), T and HT, but also less common ones, such as DHPG and CA.

**Figure 2:**
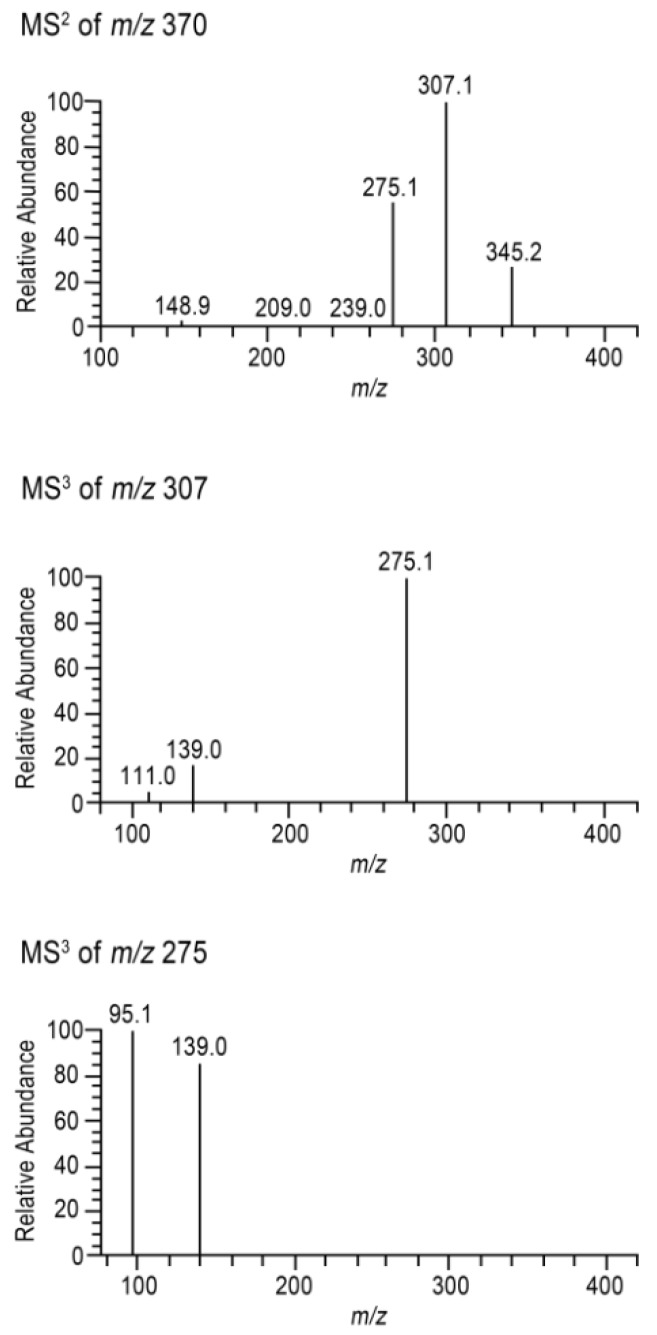
Mass spectrometry (MS^n^) spectra of the oleuropein aglycone (OA) in EVOO hydrophilic phenolic extract.

All compounds, with the exception of oleuropein aglycone isomers (OAI), were identified by a retention time comparison with pure standard solutions of the respective compound. Regarding OAIs, components were identified by ultra-high performance liquid chromatography coupled with diode-array detection and tandem mass spectrometry UHPLC-DAD-MS^n^, comparing their UV spectra and fragmentation profile with the literature data. Two components were identified as OAI (OAIa and OAIb). Their MS^n^ spectra are presented in Figure 2. Their identification was based on their [M–H]^−^ ion at *m*/*z* 377, and MS^2^ product ions at *m*/*z* 345 ([M–H–CH_3_OH]^−^), 307 ([M–H–C_4_H_6_O]^−^), and 275 ([M–H–C_4_H_6_O–CH_3_OH]^−^). Furthermore, the MS^3^ fragmentation of the ions at *m/z* 307 generates product ions at *m*/*z* 275 (−32 Da, –CH_3_OH) and 139 (−168 Da, –CH_3_OH followed by a McLafferty rearrangement), which are also characteristic product ions of OA [26]. The MS^3^ spectrum of the ions at *m*/*z* 275 also show the product ion at *m*/*z* 139, which corroborates with this product results from the consecutive losses that are described above. Finally, the UV spectra of both compounds (λ_max_ at 237 mm and 279 nm) are very similar, with the UV spectrum reported in literature for O [27].

**Figure 3:**
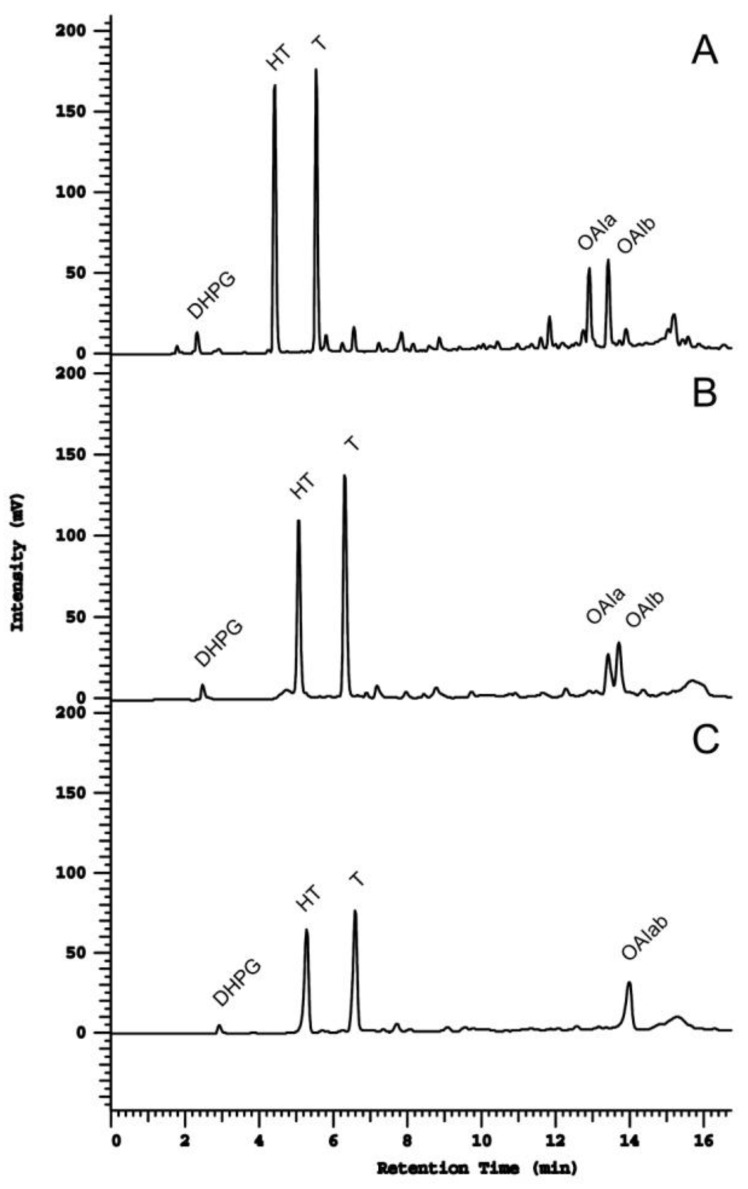
High performance liquid chromatography (HPLC)-UV chromatograms of the EVOO hydrophilic phenolic obtained with (**A**) Kinetex biphenyl column, (**B**) LiChrospher RP18 column, and (**C**) Spherisorb ODS2 column.

As for the chromatographic separation of the identified compounds, both Kinetex and LiChrospher allowed for the separation and consequent identification of six PCs in the EVOO extract (Figure 3), while only four out of six PCs were identified by using the Spherisorb column, with both OAIs eluting at a single peak. Compounds such as O and its derivatives are closely associated with health benefits, and thus, their correct identification and quantification is of high importance. Both T and HT are secoiridoid derivative compounds that are present in the olive oil polar fraction, the second one (HT) being extensively studied and well-associated with cardio-protective, anti-inflammatory and platelet aggregation, and antimicrobial and anticancer activities, among others [28]. Additionally, both T and HT are also known for their contributions to olive oil stability, due to their high antioxidant activity [29], which underlies the importance of the proper separation of these compounds, identification, and quantification.

From the fingerprinting comparison between the three tested columns (Figure 3), it is clear that an improvement in resolution was obtained with the Kinetex biphenyl column (A), mainly for OAI, which are not totally resolved with the LiChrospher column (B), and co-elute completely with the Spherisorb column (C). Additionally, a better separation of CA was obtained with this column, while no detection was observed with the Spherisorb column. Overall, the full EVOO hydrophilic phenolic profile was proven to be better resolved when using the Kinetex biphenyl column.

### 2.2. Resolution Parameters Comparison

Table 1 shows the resolution parameters that are tested for the three studied columns. The Kinetex biphenyl column showed a better performance in all the considered parameters than the other two columns for the tested peak pairs, leading to an increase in peak capacity factor (n_c_) of 31% and 35%, in relation to the LiChrospher and the Spherisorb columns, respectively. Commonly, an increase in peak capacity was achieved, increasing the stationary phase length. Donato et al. [30] described an increase of about 37% on the peak capacity when duplicating the column length. Since the gradient time was constant, the variable applied was the peak width, which was directly related to the efficiency. Therefore, it was possible to maximize the efficiency, and consequently the peak capacity, using the Kinetex biphenyl column. This decrease is similar to those that are generally achieved with higher stationary phase lengths.

Concerning the selectivity (α), which may be seen as the ability to distinguish between the analytes, according to Table 2, the best results for both peak pairs were obtained with the Kinetex biphenyl column. Special attention should be paid to the separation of OAIa and OAIb, where a selectivity of 1.0 (no separation at all) was observed when using the Spherisorb column (Figure 3C). Clearly, for these two peaks, a huge selectivity improvement was obtained when applying the Kinetex biphenyl column.

Differences in selectivity are determined by various interactions between the column stationary phase and the solute. There are several mechanisms that play an important role in column selectivity, such as hydrophobicity, hydrogen bonding, dipole–dipole, and ion exchange. Another important contributing factor for selectivity are the π–π interactions, which occur between the analyte and the stationary phases. These interactions involve the presence of aromatic rings, which in this case, are present in the biphenyl phase of the Kinetex column, and they can successfully be explored in the separation of closely related compounds, like metabolites or degradation products [31], which is in agreement with our results showing that selectivity increased when the biphenyl-phase column was used.

The retention factor (k) of HT was determined for the three tested columns. The Kinetex biphenyl column presented the best performance, with an overall retention time reduction of 22.1% (Table 2). Theoretically, for a particular substance, in this case, HT, the k factor will vary depending on the pressure used (by changing flow rate),the nature of the stationary phase, the composition of the solvent, and the temperature of the column. Since only the stationary phase was changed, the better k value observed for the Kinetex biphenyl column was due to the specific characteristics of these superficially porous particles. In this case, these particles are based on a solid core surrounded by a thin porous shell, in contrast with the fully porous particles presented on the ODS-based columns, which offer a shorter diffusion path within the working porous shell, improving the mass transfer kinetics [32].

Regarding resolution (R_s_), the best-resolved chromatograms were also obtained with the Kinetex column (Figure 2 and Table 2), with a particular improvement being observed in the case of the CA peak, which presents an acceptable resolution (Figure 2).

Peak asymmetry (A_s_) was also accessed, regarding the identified PCs for each column (Table 2). Kinetex biphenyl was the only column for which all A_s_ values lay within the range 0.8–1.4, which is considered to be suitable for better performance [33]. In opposition, the Spherisorb column presented the poorest peak shapes, with values being higher than 1.4 for DHPG and T, and lower than 0.8 for HT and OAI.

### 2.3. Method Validation

The HPLC separation method for the EVOO PCs was validated by using the Kinetex biphenyl column, and in accordance with the requirements for the development of new methods, which include accuracy, precision, selectivity, linearity and range, limit of detection (LOD) and limit of quantification (LOQ) determinations. HT and T were the PCs considered to perform this validation.

#### 2.3.1. Accuracy

The accuracy of an analytical method expresses the closeness of agreement between the value found and an expected reference value (true value), and it is measured as the percent recovery by spiking a sample with a known amount of analyte. The accuracy was determined, following the International Conference on Harmonization (ICH) recommendations [34]. Being the mean of the replicates the indication of the accuracy, and the mean recovery of the assays expected to be within 100 ± 5% at each concentration level [35,36]. The HPLC method described has revealed a good recovery of both HT and T, with values ranging from 105.1 ± 2.2 to 99.2 ± 0.8, and 105.2 ± 0.5 to 100.2 ± 1.3 for HT and HT, respectively (Table 3).

#### 2.3.2. Precision

Expressing the closeness of agreement between a series of measurements obtained from multiple sampling of the same homogeneous sample, under selected conditions, precision was measured to access the method consistency, expressed as repeatability (same operating conditions over a short period of time) [34].

Repeatability, commonly expressed as RSD (relative standard deviation) was determined for HT and T, considering the peak areas of three standard solution replicate injections with three different concentration levels (60, 150, and 260 mg/L) of the respective analyte. In order to have a good repeatability, the RSD of the replicate injections for each concentration level should not be greater than 1.5% [35]. The results showed that the testing method for HT and T quantification had a good repeatability, with the RSD being lower than 1.0%, for the specified range of concentrations.

**Figure 4:**
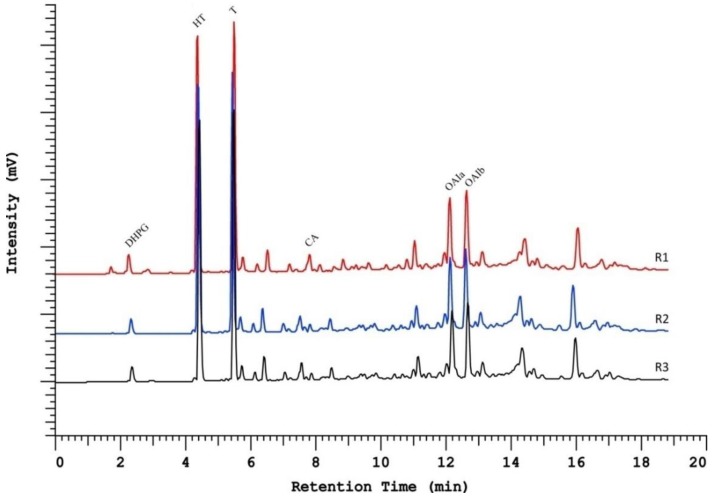
Chromatographic representation of the repeatability of the Kinetex biphenyl column, corresponding R1, R2, and R3 to different hydrophilic phenolic extractions of the same EVOO.

In order to access the method precision by evaluating repeatability, the chromatographic profiles of three hydrophilic phenolic extractions of the same EVOO sample were visually evaluated (Figure 4), revealing a method with good repeatability, since all chromatograms showed consistency in terms of retention times, peak height, and width.

#### 2.3.3. Robustness

The robustness of a method measures how a method responds to slight changes in operating parameters. The robustness of the present method was determined for the pair HT and T, and the effects of three parameters (flow rate, wavelength, and gradient method) was evaluated. Good resolutions for HT and T were obtained, even when small changes in the method occurred (Table 4).

#### 2.3.4. Linearity and Range

Linearity measures the ability to correlate test results with analyte concentrations, within a given range. The range is the interval within the analyte level that proves to be determined with precision, accuracy, and linearity [34]. The acceptance criterion is that the correlation coefficient (*r*^2^) should not be less than 0.990 for the least squares method [35]. Five concentration levels (0.5, 10, 60, 150, and 260 mg/L) were used to evaluate the linearity of the method. The analysis showed results of *r*^2^ > 0.998, proving that the method had good linearity over the specified range.

#### 2.3.5. LOD and LOQ

The LOD is the lowest amount of analyte in a sample that is able to be unequivocally detected, but not necessarily quantified, while LOQ is the lowest amount of analyte that can be quantitatively determined. For HT, the obtained results were 0.57 ± 0.07 mg/L and 1.7 ± 0.2 mg/L, for LOD and LOQ, respectively. Regarding T, the obtained LOD and LOQ were 0.6 ± 0.2 mg/L and 1.9 ± 0.7 mg/L, respectively. These results show that it is possible to quantify both HT and T at concentrations as low as 2 mg/L.

### 2.4. Method Application in a Set of Samples

**Figure 5:**
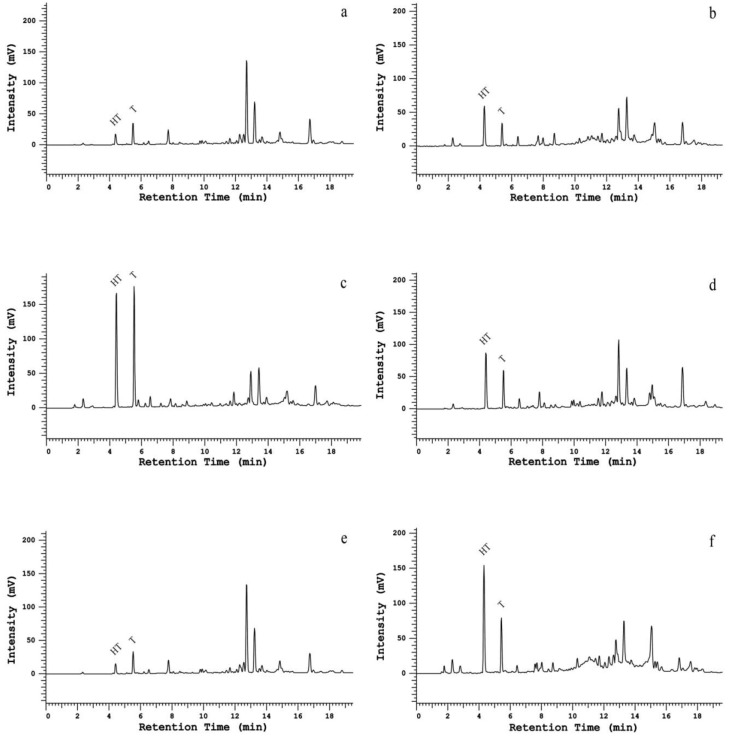
HPLC chromatograms of a set of six different EVOO samples (from (**a**–**f**)) analyzed with the Kinetex biphenyl column.

In order to further validate the proposed method for identification and quantification of PCs in EVOOs, a set of six EVOO samples from different producers were prepared and analyzed (Figure 5). Despite the different concentrations found for different samples, their profiles were quite similar, showing that a good implementation of this method was confirmed.

From Figure 5, it is possible to see that both the HT and T concentrations may vary significantly, with the EVOO quality influencing these, as stated by the health claim [7], and thus, this method was found to be a fit-for-purpose tool for the identification and quantification of PCs that were present in the hydrophilic fraction of the EVOOs.

Other methods have been proposed for the identification and quantification of hydrophilic PCs from EVOO, namely by Ricciutelli et al. [24] who applied the fully porous Synergy Polar RP column for the detection of PCs in commercial OO by the means of HPLC-DAD/MS/MS. In contrast with this study, our method was able to clearly separate and identify two OAIs, which presents a great deal of importance for quantification of “HT and its derivatives”, as specified by the health claim [7]. Additionally, a much shorter overall run time was able to be obtained in the presented method.

In sum, an efficient HPLC method to separate, identify and quantify hydrophilic PCs from EVOO was described. Considering that the HT quantity (and its derivatives) is viewed as an OO health marker, consumers have started to request this information on the EVOO bottle description. Therefore, it is of high importance to have a reliable and robust method for efficient identification and quantification of these compounds. From the three tested columns, Kinetex biphenyl proved to be the best choice to separate, identify, and quantify EVOO hydrophilic phenolic compounds. Compared to traditional fully porous C_18_ columns, the biphenyl column presented better selectivity for target analytes, shorter retentions, higher peak capacity, and better peak symmetry, leading to an overall higher resolution capacity, and thus, it was more appropriate for application in the quantification of EVOO hydrophilic PCs. The method development also proved to be within the demanded requirements for new methods, showing to be simple, rapid, accurate and robust, which can be easily applied by any laboratory for the quality control of EVOO.

## 3. Materials and Methods

### 3.1. Chemicals and Reagents

All reagents were of analytical or HPLC grade, and used as received. Methanol and acetonitrile were acquired from Merck (Darmstadt, Germany), hexane from VWR (Radnor, PA, USA), and acetic acid from Sigma-Aldrich (St. Louis, MO, USA). Double-deionized water was obtained with a Milli-Q water purification system (Millipore, Bedford, MA, USA). Standard compounds such as tyrosol, hydroxytyrosol, and oleuropein were purchased from Molekula (Gillingham, Dorset, UK), while 3,4-dihydroxyphenyl glycol and caffeic acid were purchased from Sigma-Aldrich (St. Louis, MO, USA).

### 3.2. EVOO Sample

The olive oil sample used in this work was a mixture of ‘galega’ and ‘picual’ cultivars, both supplied by a local producer from the Alentejo region (south Portugal), stored in the dark, at room temperature, in an amber glass sampling flask, until analysis.

### 3.3. HPLC Columns

Three different HPLC columns were used in the present study, namely, a Kinetex^®^ core-shell biphenyl 250 × 4.6 mm ID 5 µm column from Phenomenex (Torrance, CA, USA), and two fully porous columns, namely aLiChrospher C_18_ 250 × 4.6 mm ID 5 µm from Merck (Darmstadt, Germany) and a Spherisorb ODS2 C_18_ 250 × 4.6 mm ID 5 µm column from Waters (Milford, MA, USA).

### 3.4. Sample Preparation

For olive oil hydrophilic phenolic compounds extraction, an adaptation from A. Taamalli et al. [36] method was used: 5.00 ± 0.05 g of EVOO was weighed and dissolved in 10 mL of hexane. Then, 10 mL of a methanol/water mixture (60:40, *v*/*v*) was added, and vortexed for 30 s. Phase separation was made by centrifugation at 3500 rpm for 10 min. The extraction process was repeated three times. The hydrophilic extract was then evaporated to dryness in a rotary evaporator under low pressure at 35 °C. The final extract was dissolved in 1 mL of a methanol/water mixture (20:80, *v*/*v*, HPLC grade), and filtered through a Polytetrafluoroethylene (PTFE) 0.22 µm syringe filter before HPLC analysis.

### 3.5. HPLC Analysis

The chromatographic method used for phenolic compound detection was an adaptation from García-Villalba et al. [37] The HPLC (Merck Hitachi LaChrome, Tokyo, Japan) consisted of a L7000 interface module, a L7200 auto sampler, a L7350 column oven, a L7100 pump, an L-7420 UV detector, and was controlled with the D-7000 HSM software. The three columns described above were used for the analysis, all operating at 30 °C, and at a flow rate of 1.5 mL/min. The mobile phase consisted of water with acetic acid (0.5%) (phase A) and acetonitrile (phase B), with the following linear gradient applied: 0–10 min, 5–30% B; 10–12 min, 30–33% B; 12–17 min, 33–38% B; 17–20 min, 38–50% B; 20–23 min, 50–95% B; 23–25 min, 95% B. Finally, the B content was decreased to the initial conditions (5%) in 2 min, and the column was re-equilibrated for 10 min. A volume of 5 µL was injected. Compounds separation were monitored at a wavelength of 280 nm. Despite all of the identified peaks in this work eluting within the first 16 min, other were compounds eluted after this time (data not shown). So, the present method was conceived with a longer running time, not compromising this study, and allowing for future works on other possible compounds of interest. Running pressures never exceeded the recommended columns limits.

### 3.6. UHPLC and Tandem Mass Spectrometry Analysis

The UHPLC system consisted of a variable-loop Accela autosampler (200 vial capacity set at 15 °C), an Accela 600 LC pump, and an Accela 80 Hz photodiode array (PDA) detector (Thermo Fisher Scientific, San Jose, CA, USA). The HPLC conditions described above were the same. For MS^n^, a LCQ Fleet ion trap mass spectrometer (Thermo Finnigan, San Jose, CA, USA) was used, equipped with an electrospray ionization source operated in negative mode. The nitrogen sheath and auxiliary gas were 40 and 10 (arbitrary units), respectively. The spray voltage was 5 kV, and the capillary temperature was 350 °C. The capillary and tune lens voltages were set at −25 V and −125 V, respectively. Charge injection device (CID)-MS^n^ experiments were performed on mass-selected precursor ions in the range of *m*/*z* 100–2000. The isolation width of the precursor ions was 1.0 mass units. The collision energy was set to 35 (arbitrary units).

### 3.7. Resolution Parameters and Asymmetry Determination

Separation performance measurement was calculated using Equation (1), where peak capacity (n_c_) was calculated based on a gradient elution, t_g_ is the total gradient time, and $\bar{w}$ is the average peak width (Equation (1)).

(1) $$n_{c}=1+\left(\frac{t_{g}}{\bar{w}}\right)$$

Retention factor (k) was calculated according to Equation (2), where k is equal to the ratio of the retention time of the analyte on the column (t_R_) to the retention time of a non-retained compound (t_0_) (Equation (2)).

(2) $$k=\frac{\left(t_{R}-t_{0}\right)}{t_{0}}$$

Selectivity (α) was measured as the ratio of k of two peaks in question, representing k_1_ and k_2_ the retention factor of peak 1 and 2, respectively (Equation (3)).

(3) $$\alpha =\frac{k_{2}}{k_{1}}=\frac{t_{R2}-t_{0}}{t_{R1}-t_{0}}$$

Resolution (R_s_) was accessed according to Equation (4), where T_R1_ and T_R2_ correspond to the retention times of peak 1 and peak 2, respectively, and W_b1_ and W_b2_, the peak width of peak 1 and peak 2, respectively (Equation (4)).

(4) $$R_{s}=\frac{2\left(T_{R2}-T_{R1}\right)}{\left(W_{b1}+W_{b2}\right)}$$

Peak asymmetry (A_s_) was evaluated according to Equation (5), where A and B represent the peak width measured at 10% of the peak height, of the left and right sides of the peak, respectively (Equation (5)).

(5) $$A_{s}=\frac{B}{A}$$

### 3.8. Method Validation

The validation of the method was fully performed for both HT and T, in accordance with the requirements for new methods, which includes accuracy, precision, selectivity, robustness, linearity and range, LOD, and LOQ. For this, a calibration curve was prepared in a methanol/water solution (20:80, *v*/*v*) for both analytes, ranging from 0.5 to 260 mg/L, used for linearity and range determination. To measure accuracy, three concentration levels (60, 150, and 260 mg/L, three replicates per concentration level), covering the specified range of the method, were carried out for both HT and T. For precision determination, ISO 5725 [38] guidelines were followed, where three replicates of three different concentrations (60, 150, and 260 mg/L) of a standard solution were analyzed with the same analytical method, in the same laboratory, by the same operator and equipment, within a short period of time. The robustness of the present method was assessed by determining the critical resolution between HT and T (as standard solutions, and in EVOO derived extracts) when applying slight changes on the chromatographic conditions (flow rate, wavelength and gradient mode). The flow rate was analyzed at 1.4 mL/min and 1.6 mL/min (instead of the original 1.5 mL/min), the wavelength was analyzed at 278 nm and 282 nm (instead the normal 280 nm), and gradient conditions were set to ±2% of eluent B for all separation process. The LOD and LOQ were determined based on the standard deviation of the response, and the slope of a calibration curve [34] composed of five concentration levels (0.5, 2, 4, 6, 10 mg/L) and three replicates per concentration.

## Figures and Tables

**Table 1:** Peak capacity (n_c_), selectivity (α), retention factor (k), and resolution (R_s_), calculated for the EVOO hydrophilic phenolic extract, for the peak pairs HT–T (hidroxytyrosol–tyrosol) and OAIa–OAIb (oleuropein aglycone isomers “a” and “b”), for the three studied columns (Kinetex biphenyl, LiChrospher C_18_, and Spherisorb C_18_).

Column	n_c_	α		k	R_s_	
		HT–T	OAIa–OAIb	HT	HT–T	OAa–OAb
Kinetex biphenyl	277	1.43	1.045	1.46	5.60	2.68
LiChrospher C_18_	192	1.38	1.024	1.81	5.39	1.12
Spherisorb C_18_	179	1.37	1.0	1.94	3.91	0

**Table 2:** Peak asymmetry (A_s_) results for three tested columns, considering the identified phenolic compounds.

Identified EVOO PCs		Type of Column		
		Kinetex Biphenyl	LiChrospher C_18_	Spherisorb C_18_
DHPG	Dihydroxyphenyl glycol	1.333	2.375	1.541
HT	Hidroxytyrosol	1.25	1.143	0.615
T	Tyrosol	1.364	1.500	1.437
CA	Caffeic acid	1.2	1.353	n/d ^1^
OAIa	Oleuropein aglycone isomer a	1.333	1.555	0.468 ^2^
OAIb	Oleuropein aglycone isomer b	1.25	1.368	

^1^ n/d: not detected; ^2^ this A_s_ value corresponds to both co-eluted isomers.

**Table 3:** Accuracy of therecovery of hydroxytyrosol (HT) and tyrosol (T) at three concentration spiking levels (60, 150, and 260 mg/L) for the HPLC method proposed.

Analyte Concentration (mg/L)	% Recovery ± SD	
	HT	T
60	101.4 ± 1.6	104.2 ± 1.8
150	105.1 ± 2.2	105.2 ± 0.5
260	99.2 ± 0.8	100.2 ± 1.3

**Table 4:** Resolution (R_s_) quantification between HT (2-(3,4-dihydroxyphenyl)ethanol) and T (2-(4-hydroxyphenyl)ethanol) for different changes in the HPLC method: flow rate, wavelength, and gradient method, using the Kinetex biphenyl column.

Method Changes	R_s_ (HT-T) ± SD
No change	5.9 ± 0.2
Flow rate = 1.4 mL/min	5.91± 0.03
Flow rate = 1.6 mL/min	4.99 ± 0.06
Wavelength = 278 nm	5.6 ± 0.2
Wavelength = 282 nm	5.7 ± 0.2
Gradient method = −2% for eluent B	5.0 ± 0.1
Gradient method = +2% for eluent B	6.15 ± 0.05

## Abbreviations

CID	Charge injection device
DHPG	Dihydroxyphenyl glycol
DAD	Diode-array detection
EVOO	Extra virgin olive oil
HPLC	High performance liquid chromatography
HT	Hydroxytyrosol
ICH	International Conference on Harmonization
LOD	Limit of detection
LOQ	Limit of quantification
LLE	Liquid–liquid extraction
O	Oleuropein
OA	Oleuropeinaglycone
OAI	Oleuropeinaglycone isomers
OO	Olive oil
A_s_	Peak asymmetry
n_c_	Peak capacity
PDA	photodiode array
PC	Phenolic compounds
PTFE	Polytetrafluoroethylene
RSD	Relative standard deviation
R_s_	Resolution
k	Retention factor
RP	Reverse-phase
α	Selectivity
SPE	Solid phase extraction
MS^n^	Tandem mass spectrometry
T	Tyrosol
UV	Ultraviolet
